# Protective role of ALDH2 against acetaldehyde-derived DNA damage in oesophageal squamous epithelium

**DOI:** 10.1038/srep14142

**Published:** 2015-09-16

**Authors:** Yusuke Amanuma, Shinya Ohashi, Yoshiro Itatani, Mihoko Tsurumaki, Shun Matsuda, Osamu Kikuchi, Yukie Nakai, Shin’ichi Miyamoto, Tsunehiro Oyama, Toshihiro Kawamoto, Kelly A. Whelan, Hiroshi Nakagawa, Tsutomu Chiba, Tomonari Matsuda, Manabu Muto

**Affiliations:** 1 Department of Gastroenterology and Hepatology, Graduate School of Medicine, Kyoto University, Kyoto, 606-8507, Japan; 2 Department of Therapeutic Oncology, Graduate School of Medicine, Kyoto University, Kyoto, 606-8507, Japan; 3 Department of Surgery, Graduate School of Medicine, Kyoto University, Kyoto, 606-8507, Japan; 4 Research Center for Environmental Quality Management, Kyoto University, Otsu, 520-0811, Japan; 5 Department of Environmental Health, School of Medicine, University of Occupational and Environmental Health, Kitakyushu, 807-8555, Japan; 6 Gastroenterology Division, Department of Medicine, Abramson Cancer Center, University of Pennsylvania, Philadelphia, PA 19104, USA

## Abstract

Acetaldehyde is an ethanol-derived definite carcinogen that causes oesophageal squamous cell carcinoma (ESCC). Aldehyde dehydrogenase 2 (ALDH2) is a key enzyme that eliminates acetaldehyde, and impairment of ALDH2 increases the risk of ESCC. ALDH2 is produced in various tissues including the liver, heart, and kidney, but the generation and functional roles of ALDH2 in the oesophagus remain elusive. Here, we report that ethanol drinking increased ALDH2 production in the oesophagus of wild-type mice. Notably, levels of acetaldehyde-derived DNA damage represented by *N*^*2*^-ethylidene-2′-deoxyguanosine were higher in the oesophagus of *Aldh*2-knockout mice than in wild-type mice upon ethanol consumption. *In vitro* experiments revealed that acetaldehyde induced ALDH2 production in both mouse and human oesophageal keratinocytes. Furthermore, the *N*^*2*^-ethylidene-2′-deoxyguanosine levels increased in both *Aldh*2-knockout mouse keratinocytes and *ALDH2*-knockdown human keratinocytes treated with acetaldehyde. Conversely, forced production of ALDH2 sharply diminished the *N*^*2*^-ethylidene-2′-deoxyguanosine levels. Our findings provide new insight into the preventive role of oesophageal ALDH2 against acetaldehyde-derived DNA damage.

Oesophageal cancer is the eighth most common cancer worldwide and the sixth leading cause of cancer-related death^1^. The overall 5-year survival of patients with oesophageal cancer is poor, at 15–25%^2^. Squamous cell carcinoma is the most frequent histologic type of oesophageal cancer, particularly in East Asian countries^2^. Chronic alcohol consumption and gene variants encoding enzymes involved in alcohol metabolism are closely associated with the risk of oesophageal squamous cell carcinoma (ESCC)^3^.

Alcoholic beverages contain varying ethanol concentrations. Ingested ethanol is absorbed mainly from the duodenum and jejunum, and then metabolized to acetaldehyde by alcohol dehydrogenase in the liver^4^. Acetaldehyde is a highly reactive compound that causes DNA damage^5^,^6^. It reacts with deoxyguanosine, a substrate of DNA, and forms DNA adducts such as *N*^*2*^-ethylidene-2′-deoxyguanosine (*N*^*2*^-ethylidene-dG), *N*^*2*^-ethyl-2′-deoxyguanosine (*N*^*2*^-Et-dG) and 1,*N*^*2*^-propano-2′-deoxyguanosine^7^, which are involved in mutagenesis^8^,^9^. Among them, *N*^*2*^-ethylidene-dG is the most sensitive marker for acetaldehyde exposure^10^,^11^, and is commonly analysed to detect acetaldehyde-derived DNA damage^11^,^12^,^13^.

Aldehyde dehydrogenase 2 (ALDH2) is a mitochondrial enzyme that detoxifies acetaldehyde to acetic acid^14^. Approximately 35–45% of East Asian individuals have a single nucleotide polymorphism (G1510A) in the *ALDH2* gene^15^,^16^, which results in a Glu504Lys replacement with reduced ability to oxidize acetaldehyde. Therefore, their blood, salivary, and expiratory acetaldehyde levels after alcohol drinking are highly elevated compared with wild-type *ALDH2* homozygotes^17^,^18^,^19^. Accordingly, heavy alcohol consumers with this mutant *ALDH2* allele are at risk of ESCC because of the potential exposure of their oesophageal tissues to high amounts of acetaldehyde^20^,^21^,^22^,^23^.

Based on extensive epidemiological data, acetaldehyde associated with consumption of alcoholic beverages is defined as a ‘group 1 carcinogen’ for the oesophagus by the International Agency for Research on Cancer^24^. However, little is known about how oesophageal epithelial cells are affected by acetaldehyde. In fact, ALDH2 is produced in various tissues, including the liver, heart, and kidney^25^, but its production and functional roles in oesophageal epithelium remain elusive. In this study, we addressed the production and role of ALDH2 in oesophageal epithelium. We found that ALDH2 production was increased by acetaldehyde in oesophageal squamous epithelium and suppressed acetaldehyde-derived DNA damage.

## Results

### Effects of ethanol drinking on ALDH2 production and DNA damage in the oesophagus of Aldh2^+/+^ and Aldh2^–/–^ mice

**Figure 1:**
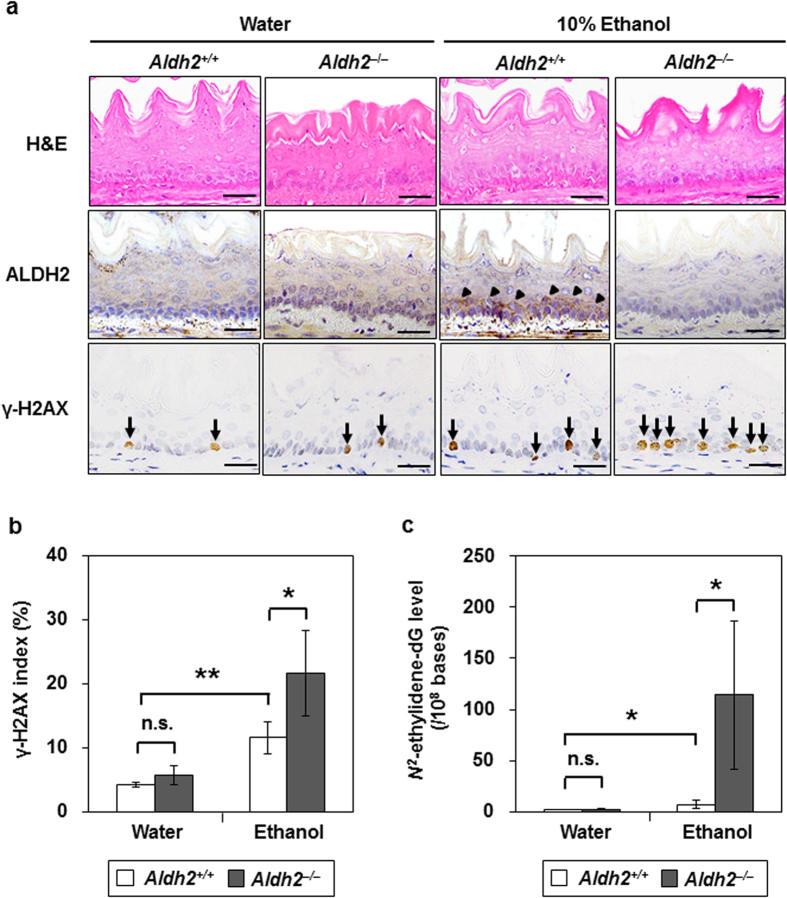
Effects of ethanol drinking in *Aldh2*^+/+^ and *Aldh2*^–/–^ mice oesophagus. The mice were allowed to drink 10% ethanol or water alone (controls) for 8 weeks. Data are presented as the mean ± SD. (**a**) Hematoxylin and eosin (H&E) and immunohistochemical staining for ALDH2 (arrowheads) and γ-H2AX (arrows). Scale bar = 50 μm. (**b**) The index of γ-H2AX staining is defined as the proportion of cells staining positive for γ-H2AX in the oesophageal basal cell layer (**p < 0.01, *p < 0.05 between the indicated groups; n = 5 in each group; n.s., not significant). (**c**) *N*^*2*^-ethylidene-dG level in the oesophagus of *Aldh2*^+/+^ and *Aldh2*^–/–^ mice with or without ethanol consumption (*p < 0.05 between the indicated groups; n = 5 in each group).

To examine whether ALDH2 was induced in the oesophagus by alcohol drinking and how it influenced alcohol-induced acetaldehyde-derived DNA damage *in vivo*, *Aldh2* wild-type (*Aldh2*^+/+^) and *Aldh*2-knockout (*Aldh2*^–/–^) mice were given 10% ethanol to drink for 8 weeks. Neither strain showed obvious histological changes in the oesophagus following ethanol drinking (Fig. 1a, upper panels). Of note, ALDH2 levels increased in the basal and parabasal cells of the oesophageal epithelium in 6 out of 10 *Aldh2*^+/+^ mice subjected to ethanol drinking, whereas they did not increase in *Aldh2*^+/+^ mice without ethanol drinking. *Aldh2*^–/–^ mice did not exhibit ALDH2 production in the presence or absence of ethanol drinking (0 of 10 mice) (Fig. 1a, middle panels; Table 1).

Next, we examined the levels of phosphorylated histone H2AX (γ-H2AX), a well-established marker of DNA damage^26^, and *N*^*2*^-ethylidene-dG in the oesophagus. γ-H2AX indexes as well as *N*^*2*^-ethylidene-dG levels in *Aldh2*^+/+^ mice with ethanol drinking were significantly higher than those without ethanol drinking (*p *< 0.001; Fig. 1b), (*p* = 0.02; Fig. 1c), respectively. Moreover, both levels were significantly elevated in *Aldh2*^–/–^ mice with ethanol drinking compared to *Aldh2*^+/+^ mice with ethanol drinking (*p *= 0.02; Fig. 1b), (*p* = 0.03; Fig. 1c), respectively, although there was no significant difference between *Aldh2*^–/–^ and *Aldh2*^+/+^ mice without ethanol drinking (Fig. 1b,c). Thus, ethanol drinking induced the production of ALDH2 in the oesophagus, and acetaldehyde-derived oesophageal DNA damage was enhanced in the absence of *Aldh2* gene expression.

### Effects of acetaldehyde on DNA damage and ALDH2 production in human oesophageal keratinocytes

**Figure 2:**
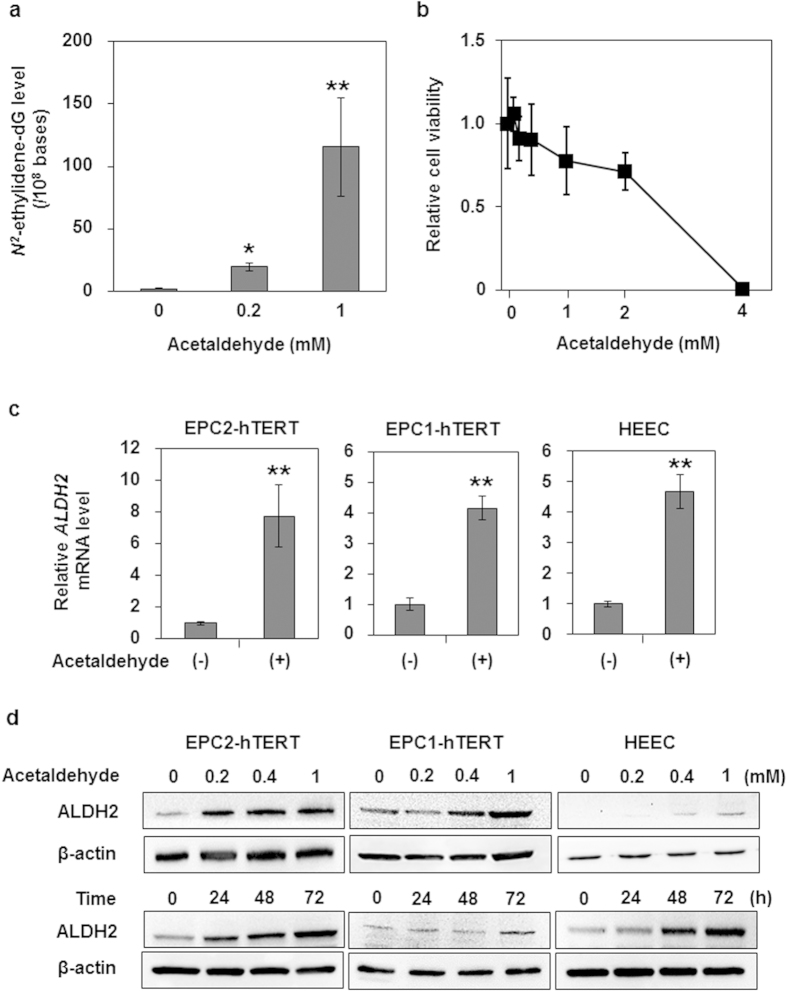
Effects of acetaldehyde treatment on human oesophageal keratinocytes. Data are presented as the mean ± SD. (**a**) *N*^*2*^-ethylidene-dG levels in DNA isolated from EPC2-hTERT cells treated with the indicated concentrations of acetaldehyde for 72 h (**p < 0.01, *p < 0.05 vs. the cells treated with 0 mM acetaldehyde; n = 3). (**b**) Cell viability of EPC2-hTERT cells treated with various concentrations of acetaldehyde for 72 h. The ratio of viable cells is expressed relative to the cells treated with 0 mM acetaldehyde (n = 6 for each acetaldehyde concentration). (**c**) ALDH2 mRNA expression levels in EPC2-hTERT, EPC1-hTERT, and HEEC cells after treatment with acetaldehyde (1 mM) for 72 h. The mRNA levels for the *ALDH2* gene relative to the cells treated with 0 mM acetaldehyde were determined by quantitative real-time reverse transcription PCR; the gene for β-actin served as an internal control (**p < 0.01 vs. the cells treated with 0 mM acetaldehyde; n = 3). (**d**) ALDH2 protein production levels in EPC2-hTERT, EPC1-hTERT, and HEEC cells after treatment with the indicated concentrations of acetaldehyde for 72 h (upper panels) or with acetaldehyde (0.2 mM) for the indicated time points (lower panels). ALDH2 protein production levels were determined by western blotting, and β-actin served as a loading control.

To examine how acetaldehyde affects oesophageal keratinocytes, we treated human oesophageal keratinocytes immortalized with human telomerase reverse transcriptase (hTERT; EPC2-hTERT cells) with acetaldehyde and assessed DNA damage and cell viability. As shown in Fig. 2a, acetaldehyde induced DNA adduct formation in a dose-dependent manner at doses of less than or equal 1 mM that did not induce substantive cell death (Fig. 2b).

Next, we hypothesized that ALDH2 levels would be increased in oesophageal keratinocytes by acetaldehyde administration, as an autonomous cytoprotective mechanism. To test this hypothesis, we treated three independent human oesophageal keratinocyte cell lines (EPC2-hTERT, EPC1-hTERT, and HEEC) carrying wild-type *ALDH2* with acetaldehyde. We found that each of these cell lines displayed enhanced expression of ALDH2 mRNA and protein levels upon stimulation with acetaldehyde in times and dose-dependent manners (Fig. 2c,d). These data indicate that acetaldehyde directly increased ALDH2 production in oesophageal epithelial cells.

### Effects of depletion of ALDH2 on acetaldehyde-induced DNA damage

**Figure 3:**
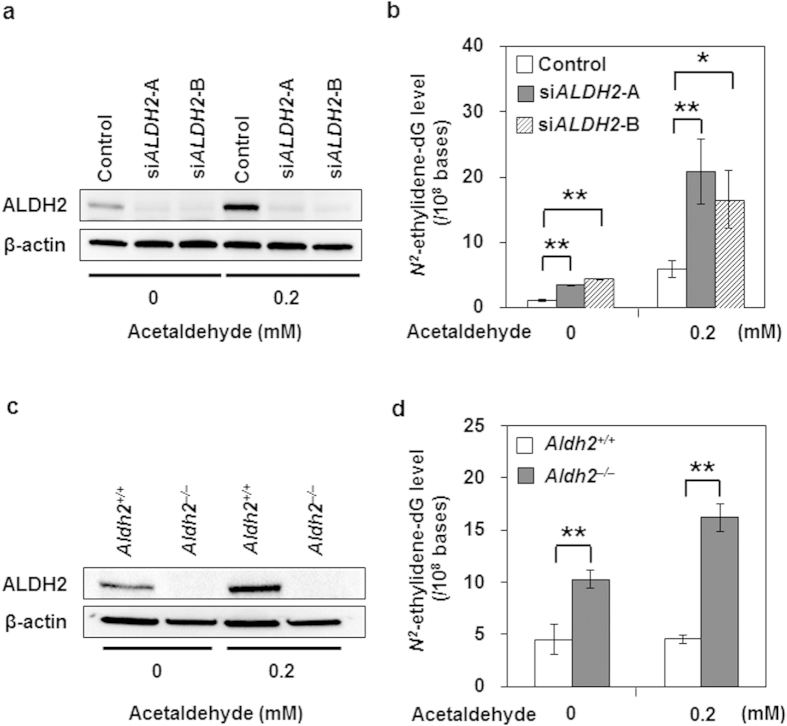
Facilitation of acetaldehyde-derived DNA damage caused by ALDH2 depletion in human and mouse oesophageal keratinocytes. EPC2-hTERT cells were treated with 0 or 0.2 mM acetaldehyde for 72 h following transfection with siRNA targeting *ALDH2* mRNA translation (*siALDH2*). Oesophageal keratinocytes isolated from *Aldh2*^*+/+*^ or *Aldh2*^–/–^ mice were treated with 0 or 0.2 mM acetaldehyde for 72 h. Data are presented as the mean ± SD. (**a**) Western blotting showing the effect of RNA interference for *ALDH2* on EPC2-hTERT cells; β-actin served as a loading control for whole cell lysates. (**b**) *N*^*2*^-ethylidene-dG levels in EPC2-hTERT cells treated with or without si*ALDH2*-A or si*ALDH2*-B in 0 or 0.2 mM acetaldehyde (**p < 0.01, *p < 0.05 between pairs of indicated groups; n = 3 in each group). (**c**) Western blotting showing ALDH2 levels in mouse oesophageal keratinocytes in 0 or 0.2 mM acetaldehyde; β-actin served as a loading control. (**d**) *N*^*2*^-ethylidene-dG level in oesophageal keratinocytes isolated from *Aldh2*^*+/+*^ and *Aldh2*^*–/–*^ mice in 0 or 0.2 mM acetaldehyde (**p < 0.01 between pairs of indicated groups; n = 5 in each group).

To determine the functional role of ALDH2 in human oesophageal keratinocytes, we knocked down *ALDH2* expression by small interfering RNA (siRNA) in EPC2-hTERT cells. *ALDH2*-knockdown by siRNA (si*ALDH2*-A and si*ALDH2*-B) reduced ALDH2 protein production, whereas a nonsilencing siRNA sequence did not (Fig. 3a). The basal *N*^*2*^-ethylidene-dG level was significantly higher in both *ALDH2*-knockdown cells than in the control cells (*p *< 0.001 and *p *< 0.001, respectively) (Fig. 3b). Furthermore, acetaldehyde treatment markedly increased the *N*^*2*^-ethylidene-dG level in both *ALDH2*-knockdown cells compared with control cells (*p *= 0.007 and *p *= 0.016, respectively) (Fig. 3b).

To confirm the enhancement of acetaldehyde-derived DNA damage by ALDH2 impairment, we also conducted *ex vivo* experiments using mouse oesophageal keratinocytes isolated from *Aldh2*^+/+^ and *Aldh2*^–/–^ mice and treated with acetaldehyde. ALDH2 production was absent in *Aldh2*^–/–^ oesophageal keratinocytes irrespective of acetaldehyde treatment, whereas it was increased by this treatment in keratinocytes of *Aldh2*^+/+^ mice (Fig. 3c). Consistent with the results of the siRNA experiments (Fig. 3b), the basal *N*^*2*^-ethylidene-dG level was significantly higher in *Aldh2*^–/–^ cells than in *Aldh2*^+/+^ cells (*p *< 0.001) (Fig. 3d). Moreover, after acetaldehyde treatment, the *N*^*2*^-ethylidene-dG level was significantly elevated in *Aldh2*^–/–^ cells, whereas it was not increased in *Aldh2*^+/+^ cells (*p *< 0.001) (Fig. 3d). These results collectively suggest that endogenous ALDH2 plays a role in preventing acetaldehyde-derived DNA damage in oesophageal keratinocytes.

### Effects of ALDH2 overexpression on acetaldehyde-derived DNA damage

**Figure 4:**
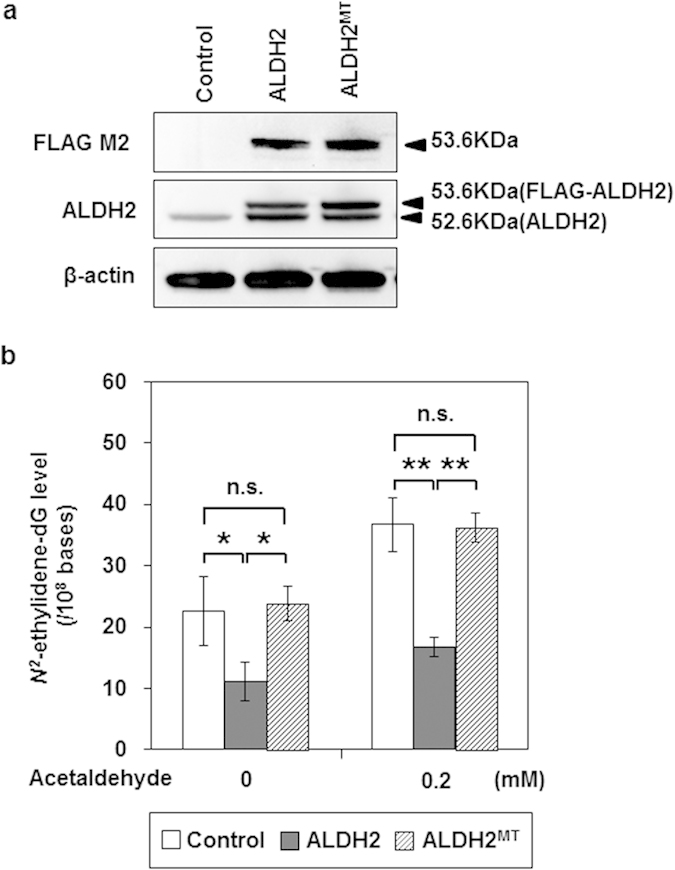
Reduction of acetaldehyde-derived DNA damage by overexpression of ALDH2 in EPC2-hTERT cells. EPC2-hTERT cells were transduced with lentiviral pLEX-ALDH2 or pLEX-ALDH2^MT^ vectors to establish EPC2-ALDH2 or EPC2-ALDH2^MT^ cells, respectively, which stably overexpressed wild-type or mutant *ALDH2*. As control cells, we transduced EPC2-hTERT cells with a lentiviral control vector bearing a *FLAG*-tag without an *ALDH2* coding site. Data are presented as the mean ± SD. (**a**) Western blotting showing overproduction of *FLAG*-tagged ALDH2 protein (53.6 kDa) and non-*FLAG*-tagged ALDH2 (52.6 kDa) in EPC2-ALDH2 and EPC2-ALDH2^MT^ cells using antibodies to FLAG M2 and ALDH2; β-actin served as a loading control. (**b**) *N*^*2*^-ethylidene-dG levels in EPC2-ALDH2, EPC2-ALDH2^MT^, and control cells treated with 0 or 0.2 mM acetaldehyde for 72 h (**p < 0.01, *p < 0.05 between the indicated groups; n = 3 in each group; n.s., not significant).

Finally, we examined whether *ALDH2* overexpression would decrease acetaldehyde-derived DNA damage. The control EPC2-hTERT cells, transduced with a lentiviral control vector bearing a *FLAG*-tag but lacking the *ALDH2* coding site, showed production of endogenous ALDH2 protein (52.6 kDa). EPC2-hTERT cells stably overexpressing wild-type *ALDH2* or mutant *ALDH2*, designated as EPC2-ALDH2 or EPC2-ALDH2^MT^ cells respectively, showed substantial production of both exogenous *FLAG*-tagged ALDH2 protein (53.6 kDa) and endogenous ALDH2 protein (52.6 kDa) (Fig. 4a). Basal *N*^*2*^-ethylidene-dG levels were significantly lower in EPC2-ALDH2 cells than in either control cells (*p *= 0.032) or EPC2-ALDH2^MT^ cells (*p *= 0.02) (Fig. 4b). Most importantly, the *N*^*2*^-ethylidene-dG production induced by acetaldehyde treatment was also significantly smaller in EPC2-ALDH2 cells than in control (*p *< 0.001) or EPC2-ALDH2^MT^ cells (*p *= 0.001) (Fig. 4b).

## Discussion

Here, we found that ethanol drinking increased ALDH2 production in the oesophagus of *Aldh2*^*+/+*^ mice. Compared with *Aldh2*^*+/+*^ mice, the *Aldh2*^–/–^ mice displayed greatly enhanced acetaldehyde-derived DNA damage in their oesophagus. We also found that acetaldehyde directly enhanced ALDH2 production in both mouse and human oesophageal keratinocytes *in vitro*, and that acetaldehyde-derived DNA damage was augmented in both human *ALDH2*-knockdown keratinocytes and *Aldh2*^*–/–*^ mouse keratinocytes. Furthermore, overexpression of wild-type *ALDH2*, but not mutant *ALDH2*, reduced acetaldehyde-derived DNA damage in human keratinocytes. These data indicate that oesophageal ALDH2 plays a protective role in reducing acetaldehyde-derived DNA damage in keratinocytes.

Our *in vivo* study revealed that ethanol drinking induced ALDH2 production in the basal and parabasal layers of the mouse oesophagus. It is controversial whether ALDH2 protein is produced in the oesophagus. Yin *et al.* reported that agarose isoelectric focusing did not show ALDH2 expression in human oesophageal mucosa^27^. By contrast, in an immunohistochemistry study, Morita *et al.* found that ALDH2 was produced in the oesophageal epithelium and that the expression levels were closely associated with the patients’ drinking habits^28^. Our data are in agreement with those reported by Morita *et al.* Moreover, we demonstrated that oesophageal ALDH2 production was induced by acetaldehyde exposure in human and mouse oesophageal keratinocytes. These results suggest that the increased oesophageal ALDH2 levels induced by ethanol drinking are triggered by the direct exposure of oesophageal mucosal cells to acetaldehyde rather than to ethanol *per se*.

To determine the role of oesophageal ALDH2 in acetaldehyde-derived DNA damage, we conducted *in vitro* and *ex vivo* experiments in which the same amount of acetaldehyde was administered to both human and mouse oesophageal keratinocytes with genetic modifications to ALDH2 production levels, and found a strong negative association between the extent of acetaldehyde-derived DNA damage and ALDH2 levels. These results indicate that oesophageal ALDH2 might act genoprotectively for acetaldehyde as an autonomous defence response to acetaldehyde exposure. Thus, DNA damage might be caused by acetaldehyde exposure that exceeds the innate defence capacity of oesophageal keratinocytes.

Immunohistochemical analysis of γ-H2AX in our *in vivo* study showed that DNA damage was accumulated at the basal layer of the oesophageal epithelium in *Aldh2*^*–/–*^ mice following ethanol drinking. Acetaldehyde-derived DNA adducts are known to induce DNA–DNA crosslinking, which leads to gene mutations and double-strand DNA breaks, and ultimately to the induction of γ-H2AX expression^6^,^29^. This result indicates that the basal cells in the oesophageal epithelium might be susceptible to DNA damage induced by acetaldehyde.

In our *in vivo* study, obvious histologic abnormalities were not found in *Aldh2*^–/–^ mice with ethanol drinking for 8 weeks, although DNA adducts levels highly increased in those mice. Similarly, *N*^*2*^-ethylidene-dG levels increased in acetaldehyde-treated ALDH2-knockdown cells; however, those cells were not transformed in the present study. Moreover, proliferative and migrative activities of those cells were not influenced by acetaldehyde treatment (supplementary Figure S1). Accordingly, it is still unclear whether DNA adducts are the direct initiators of alcohol-related carcinogenesis in the oesophagus.

In our study, overexpression of wild-type ALDH2 in the oesophageal mucosal cells reduced acetaldehyde-derived DNA damage. Recently, ALDH2 is reported as an important mediator of endogenous cytoprotection in the heart when it is subjected to ischemia^30^,^31^. In addition, overexpression of the *ALDH2* transgene ameliorates acute cardiac toxicity of ethanol^32^ as well as acetaldehyde-induced cell injury in human cardiac myocytes or umbilical vein endothelial cells^33^,^34^. These findings support the notion that ALDH2 induction or activation might have a preventive potential to alcohol- or acetaldehyde-induced cytotoxicity in the oesophagus.

A limitation of this study is that we could not demonstrate ALDH2 enzymatic activity in oesophageal tissues and keratinocytes. To show such activity, we conducted experiments to quantify the reduction of nicotinamide adenine dinucleotide production by ALDH2 as described previously^35^. We could measure the ALDH activity in mouse liver (0.9 ± 0.22 nmol/min/mg protein); however, its activity in the mouse oesophagus and human oesophageal keratinocytes were undetectable. Because the ALDH2 expression level determined by western blotting was very low in the mouse oesophagus compared with the liver (Supplement Figure S2), the above experiments might not be able to determine ALDH activity in the mouse oesophagus. Additional research is warranted to elucidate the relationship between ALDH2 expression level and its activity.

In conclusion, we have demonstrated here that ALDH2 production in oesophageal keratinocytes is induced by acetaldehyde and that oesophageal ALDH2 acts cytoprotectively as a local defence mechanism against acetaldehyde-derived DNA damage.

## Methods

### Mouse preparation and alcohol drinking

Six-week-old male *Aldh2*^*–/–*^ mice^36^, backcrossed with C57BL/6 mice, were obtained from the Department of Environmental Health, University of Occupational and Environmental Health (Kitakyushu, Japan). Age-matched control C57BL/6 male mice, which carry the wild-type *Aldh2*, were purchased from Charles River Laboratories Japan Inc. (Yokohama, Japan). All experiments conformed to the relevant regulatory standards and were approved by the Institutional Animal Care and Use Committee of Kyoto University (Med Kyo 14522). The genotype of *Aldh2* was determined by polymerase chain reaction (PCR) amplification as described^36^. *Aldh2*^*+/+*^ and *Aldh2*^–/–^ mice were allowed to drink water with or without 10% ethanol supplementation freely for 8 weeks as described^37^. Mice were euthanized painlessly under anesthesia with diethyl ether inhalation followed by cervical dislocation. Oesophageal tissues were collected in 4% Paraformaldehyde Phosphate Buffer Solution (Wako Pure Chemical Industries, Ltd., Osaka, Japan) for immunohistochemistry (n = 10 each group) or frozen in liquid nitrogen and stored at –80 °C for analysis of DNA adducts (n = 5 each group).

### Oesophageal keratinocyte culture and treatments

Primary mouse oesophageal epithelial cells (keratinocytes) were isolated from C57BL/6 mice and grown as described^38^. Two independent nontransformed human oesophageal keratinocyte cell lines, EPC1-hTERT and EPC2-hTERT, were obtained from the Cell Culture Core of the Center for Molecular Studies in Digestive and Liver Diseases at the University of Pennsylvania^39^,^40^. They were cultured in Keratinocyte Serum-Free Media (Life Technologies Corp., Carlsbad, CA, USA), supplemented with 40 μg/mL of Bovine Pituitary Extract, 1 ng/mL Epidermal Growth Factor, and 1% penicillin/streptomycin. A primary human oesophageal epithelial cell line, HEEC^41^, was purchased from ScienCell Research Laboratories (Carlsbad, CA, USA) and cultured in Epithelial Cell Medium-2 (EpiCM-2). The *ALDH2* genotype of human oesophageal keratinocytes was assessed using SmartAmp ALDH2 Typing Kits (DNAFORM, Yokohama, Japan). Cells were treated with acetaldehyde (0–4 mM) (Sigma-Aldrich, St. Louis, MO, USA) for 0–72 h. Cell viability was determined by sulfonated tetrazolium salt WST-1 assays according to the manufacturer’s instructions (Roche Applied Science, Penzberg, Germany).

### Generation of the ALDH2 transgene construct

To establish a stably transfected cell line expressing *ALDH2*, we produced a lentiviral plasmid vector. The *ALDH*2 open reading frame was PCR-amplified using Mammalian Gene Collection Human ALDH2 Sequence-Verified cDNA (Clone ID: 5477768) (Thermo Fisher Scientific Inc., Waltham, MA, USA) as a template with PrimeSTAR Max DNA Polymerase (Takara Bio Inc., Otsu, Japan) and ALDH2-Forward (5′–AAAAAGAATTCATGTTGCGCGCTGCCGCC–3′) and ALDH2-Reverse (5′–AAAAACTCGAGTTATGAGTTCTTCTGAGG–3′) primers. The amplified *ALDH2* fragment was ligated into the EcoRI and XhoI sites of a pCMV-Tag2B vector (Stratagene Corp., La Jolla, CA, USA) by Ligation Mix (Takara Bio Inc.), resulting in the creation of pCMV-ALDH2. pCMV-ALDH2 was cut by NheI and XhoI to include the coding site for *FLAG*-tagged ALDH2. The lentiviral plasmid pLEX-MCS vector^42^ (Thermo Fisher Scientific Inc.) was cut by SpeI and XhoI. The site of *FLAG*-*ALDH2* was cloned into a pLEX-MCS vector. The construct sequence was confirmed by sequencing, and designated as the pLEX-ALDH2 plasmid vector. To produce recombinant lentiviruses, pMD2.G and psPAX2 (Addgene, Cambridge, MA, USA) were cotransfected with the pLEX-ALDH2 vector into HEK293T cells using Lipofectamine LTX (Life Technologies Corp.). Finally, a recombinant *ALDH2*-coding lentivirus was transduced into EPC2-hTERT cells, and the cells were selected by puromycin (1 μg/ml) (Sigma-Aldrich). We constructed a control vector bearing a *FLAG*-tag and lacking the *ALDH2* coding site. We also constructed a lentiviral plasmid vector including a mutant *ALDH2* with a nucleotide substitution (G1510A; Glu504Lys) in exon 12 of the *ALDH2* locus—this point mutation causes catalytic deficiency of human ALDH2^15^—using the PrimeSTAR Mutagenesis Basal Kit (Takara Bio Inc.) according to the manufacturer’s protocol, and designated this as the pLEX-ALDH2^MT^ vector. The mutated primers were as follows: forward (5′–ATACACTAAAGTGAAAACTGTCACAG–3′) and reverse (5′–TTTCACTTTAGTGTATGCCTGCAGCC–3′).

### Immunohistochemistry

Immunohistochemistry was performed as described^43^. Primary antibodies and the titers used were as follows: rabbit polyclonal anti-ALDH2 (Ag7452; 15310-1-AP; Proteintech Group, Inc., Chicago, IL, USA; 1:500); rabbit monoclonal anti-phospho-histone H2AX (Ser139; 20E3; Cell Signaling Technology, Inc., Danvers, MA, USA; 1:480). Immunostained tissues were assessed using a Keyence BIOREVO BZ-9000 microscope (Keyence Corp., Osaka, Japan). For the staining of ALDH2, we defined the sample as positive when more than 50% of the cells were stained with anti-ALDH2 in the basal and parabasal layer of the oesophageal epithelium together. We present the ratio of cells positive for phosphorylated histone H2AX (γ-H2AX) to the total number of cells, determined using a Hybrid Cell Count BZ-H2C system (Keyence Corp.), as a staining index.

### DNA isolation, digestion, and quantification of N^2^-ethylidene-dG

DNA was isolated from tissue specimens or cell pellets as described^11^. To quantify acetaldehyde-derived DNA damage, we measured the *N*^*2*^-ethylidene-dG level in the oesophagus of mice and cultured cells as described^37^. Briefly, NaBH_3_CN (100 mM) (Sigma-Aldrich), a reducing reagent, was added to DNA samples. This converts *N*^*2*^-ethylidene-dG to stable *N*^*2*^-Et-dG. As the endogenous *N*^*2*^-Et-dG level in tissues is extremely low, the *N*^*2*^-Et-dG level that is converted from *N*^*2*^-ethylidene-dG indicates the endogenous *N*^*2*^-ethylidene-dG level^10^. The DNA adduct standard, *N*^*2*^-Et-dG, and its stable isotope, [U-^15^N_5_]-labeled *N*^*2*^-Et-dG, were synthesized as described^11^. DNA samples were digested as described^11^, and subjected to liquid chromatography tandem mass spectrometry (LC/MS/MS). LC/MS/MS analyses were performed using a Shimadzu LC system (Shimadzu Corp., Kyoto, Japan) interfaced with a Quattro Ultimo triple-stage quadrupole mass spectrometer or an ACQUITY UPLC H-Class system interfaced with a XEVO-TSQ triple-stage quadrupole mass spectrometer (Waters Corp., Milford, MA, USA) as reported^11^. Shim-pack XR-ODS columns (3.0 × 75 mm, 2.2 μm; Shimadzu Corp.) or ACQUITY UPLC BEH C18 columns (2.1 × 100 mm, 1.7 μm; Waters Corp.) were used to separate the samples.

### TaqMan gene expression assays

RNA was isolated using RNeasy Plus Mini Kits (QIAGEN Inc., Hilden, Germany) and cDNA was synthesized using PrimeScript RT reagent kits (Takara Bio Inc.) according to the manufacturer’s instructions. Real-time reverse-transcription PCR was done with TaqMan Gene Expression Assays (Life Technologies Corp.) for ALDH2 (Assay ID; Hs01007998_m1) and for β-actin (Assay ID; Hs99999903_m1) using a LightCycler 480 Instrument II (Hoffmann-La Roche Ltd, Basel, Switzerland) as described^44^. All PCR reactions were performed in triplicate. The relative mRNA expression level was normalized to that of β-actin as an internal control.

### Western blot analysis

Whole cell lysates were prepared as described^45^. The denatured protein samples (20 μg) were fractionated on Any kD Mini-PROTEAN TGX Precast Gels (Bio-Rad Laboratories, Inc., Hercules, CA, USA). Primary antibodies and the titers used for western blotting were as follows: goat polyclonal anti-ALDH2 (N-14) (sc-48838; Santa Cruz Biotechnology, Inc., Santa Cruz, CA, USA; 1:1000); mouse monoclonal anti-FLAG M2 (F1805; Sigma-Aldrich; 1:1000); rabbit monoclonal anti-β-actin (13E5; Cell Signaling Technology, Inc.; 1:2000). These were then reacted with the appropriate horseradish peroxidase-conjugated secondary antibody (GE Healthcare, Little Chalfont, UK). The signal was visualized using an Immobilon Western Chemiluminescent Horseradish Peroxidase Substrate (Merck Millipore, Darmstadt, Germany) and was exposed using a ChemiDoc XRS system equipped with Image Lab software (Bio-Rad Laboratories, Inc.).

### RNA interference

To suppress endogenous *ALDH2* induction, FlexiTube siRNA (QIAGEN Inc.) for *ALDH2* (two independent sequences: *ALDH2*-A, SI03059973; *ALDH2*-B, SI03087070) were employed. AllStars Negative Control (1027280) (QIAGEN Inc.) was used as a nonsilencing control. The siRNAs (10 nM) were transfected transiently using HiPerFect transfection reagent (QIAGEN Inc.), according to the manufacturer’s instructions.

### Statistical analysis

Data are presented as the mean ± standard deviation. The data were first tested for normality of distribution. The differences between two groups were analysed using two-tailed paired Student’s *t* tests for equal variance data or using Welch’s *t* test for the unequal variance data presented in Figs 1b,c and 2a,c and 3d. Student’s *t* test followed by the Bonferroni post hoc test was used to analyse the data presented in Fig. 3b. One-way analysis of variance (ANOVA) followed by Tukey’s post hoc analysis was used to analyse the data shown in Fig. 4b. Fisher’s exact test was used to assess the relationship between ALDH2 expression and drinking ethanol in *Aldh2*^*+/+*^ mice (data presented in Table 1). Statistical analyses were performed using SPSS software (version 17; IBM Corp., Armonk, NY, USA). *P*-values < 0.05 were considered significant.

## Additional Information

**How to cite this article**: Amanuma, Y. *et al.* Protective role of ALDH2 against acetaldehyde-derived DNA damage in oesophageal squamous epithelium. *Sci. Rep.* **5**, 14142; doi: 10.1038/srep14142 (2015).

## Supplementary Material

Supplementary Information

## Figures and Tables

**Table 1:** ALDH2 protein production in the oesophagus of *Aldh2* ^+/+^ and *Aldh2* ^–/–^ mice treated with or without ethanol drinking.

Groups	**ALDH2 level**	
	**Negative**	**Positive**
*Aldh2*^*+/+*^ mice		
Water	10	0
Ethanol	4	6
*Aldh2*^*–/–*^ mice		
Water	10	0
Ethanol	10	0

We defined positive of ALDH2 protein levels when more than 50% of the cells were stained with anti-ALDH2 antibody in the basal and parabasal layers of the oesophageal epithelium. Ethanol drinking induced ALDH2 in the oesophageal epithelium in 6 out of 10 *Aldh2*^+/+^ mice, whereas there was no induction in the water group of *Aldh2*^+/+^ mice (*p *= 0.011 by Fisher’s exact test).
