# Supplementary material for: Protective role of ALDH2 against acetaldehyde-derived DNA damage in oesophageal squamous epithelium
Source: Sci Rep. 2015 Sep 16;5:14142. doi: 10.1038/srep14142 (PMC4570974; doi:10.1038/srep14142)
Supplement: Supplementary Information [file srep14142-s1.pdf]

Protective role of ALDH2 against acetaldehyde-derived DNA damage in oesophageal squamous epithelium

Yusuke Amanuma, Shinya Ohashi, Yoshiro Itatani, Mihoko Tsurumaki, Shun Matsuda, Osamu Kikuchi, Yukie Nakai, Shin'ichi Miyamoto, Tsunehiro Oyama, Toshihiro Kawamoto, Kelly A. Whelan, Hiroshi Nakagawa, Tsutomu Chiba, Tomonari Matsuda, Manabu Muto

Supplementary Method:

Cell Migration Assay

Cell migration assays were performed using CytoSelect™ 24-Well Cell Migration Assay Kit (8 µm, Fluorometric Format) (CELL BIOLABS, INC., San Diego, CA, USA) according to the manufacturer's instructions. In brief, the lower chamber was filled with 0.5 ml of Keratinocyte Serum-Free Media (Life Technologies Corp.), supplemented with 40 µg/mL of Bovine Pituitary Extract, 1 ng/mL Epidermal Growth Factor, and 1% penicillin/streptomycin, and 0.3 ml cell suspension in Keratinocyte Basal Media (Lonza Walkersville, Inc., Walkersville, MD, USA) containing no growth factors, cytokines, or supplements were plated in the upper insert in triplicate wells and incubated at 37 °C for 18 h. Then migratory cells were dissociated from membrane by the addition of Cell Detachment Buffer to the lower chamber. Migratory cells were lysed and quantified using CyQuan® GR Fluorescent Dye at 480 nm/520 nm.

S1

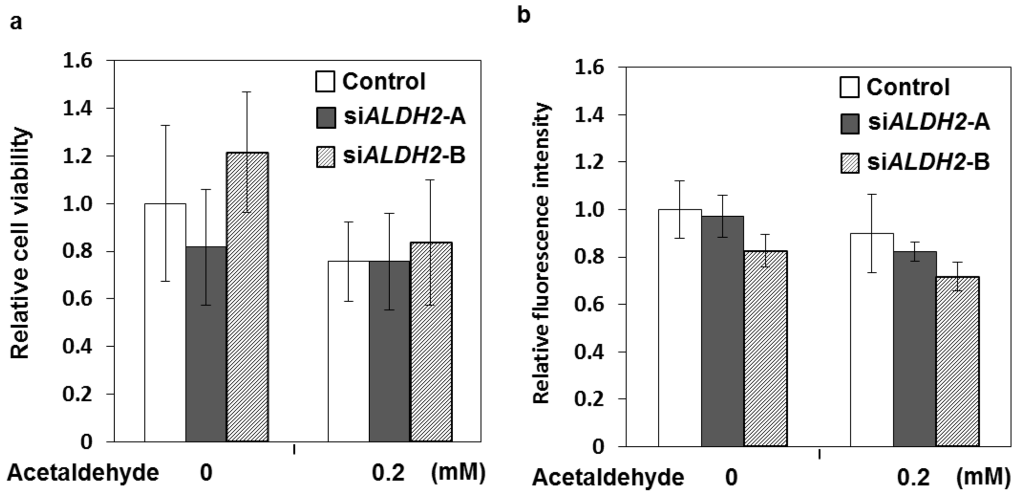

Figure S1. Cell viability and migrative activity of EPC2-hTERT cells treated with or without acetaldehyde (0.2 mM) for 48 h following transfection with siRNA targeting *ALDH2* mRNA translation (*siALDH2*). Data are presented as the mean ± SD. The proliferative and migrative activities of *ALDH2*-knockdown cells were not influenced by acetaldehyde treatment (a) The ratio of viable cells was determined by WST-1 assay, and it is expressed relative to the control cells treated with 0mM acetaldehyde (n = 4). (b) The ratio of migratory cells was determined by migration assay, and it is expressed relative to the control cells treated with 0mM acetaldehyde (n = 4).

S2

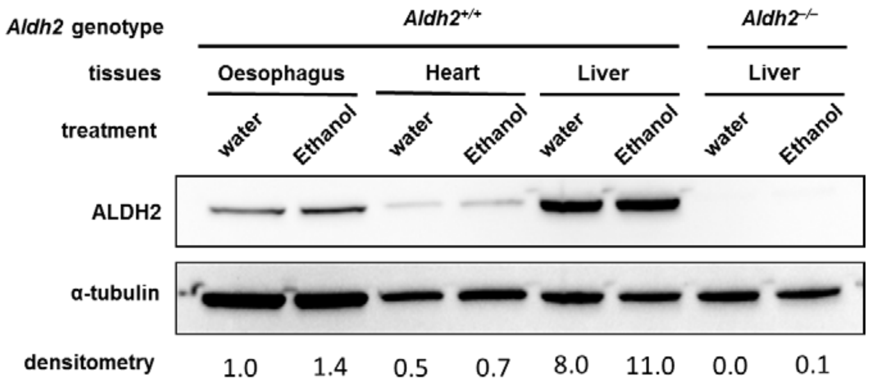

Figure S2. ALDH2 protein production levels in the oesophagus, heart and liver of *Aldh2*$^{+/+}$ and in the liver of *Aldh2*$^{-/-}$ mice with or without ethanol drinking for 8 weeks. ALDH2 protein production levels were determined by western blotting, and α-tubulin was used as a loading control. Densitometry ratios of ALDH2/α-tubulin were calculated and recorded.
